# Clinical Characteristics and Outcomes of Severe and Critical Patients With 2019 Novel Coronavirus Disease (COVID-19) in Wenzhou: A Retrospective Study

**DOI:** 10.3389/fmed.2020.552002

**Published:** 2020-09-04

**Authors:** Song-Zan Qian, Wan-dong Hong, Lingjie-mao, Chenfeng-lin, Zhendong-fang, Jing-Ye Pan

**Affiliations:** ^1^Department of Intensive Care Unit, The First Affiliated Hospital of Wenzhou Medical University, Wenzhou, China; ^2^Wenzhou Key Laboratory of Critical Care and Artificial Intelligence, Wenzhou, China; ^3^Department of Gastroenterology, The First Affiliated Hospital of Wenzhou Medical University, Wenzhou, China; ^4^School of the First Clinical Medical Sciences, Wenzhou Medical University, Wenzhou, China

**Keywords:** COVID-19, infection, severity, critically ill, outcome

## Abstract

Information about severe cases of 2019 novel coronavirus disease (COVID-19) infection is scarce. The aim of this study was to report the clinical characteristics and outcomes of severe and critical patients with confirmed COVID-19 infection in Wenzhou city. In this single-centered, retrospective cohort study, we consecutively enrolled 37 RT-PCR confirmed positive severe or critical patients from January 28 to February 16, 2020 in a tertiary hospital. Outcomes were followed up until 28-day mortality. Fifteen severe and 22 critical adult patients with the COVID-19 infection were included. Twenty-six (68.4%) were men. Echocardiography data results suggest that normal or increased cardiac output and diastolic dysfunction are the most common manifestations. Compared with severe patients, critical patients were older, more likely to exhibit low platelet counts and high blood urea nitrogen, and were in hospital for longer. Most patients had organ dysfunction during hospitalization, including 11 (29.7%) with ARDS, 8 (21.6%) with acute kidney injury, 17 (45.9%) with acute cardiac injury, and 33 (89.2%) with acute liver dysfunction. Eighteen (48.6%) patients were treated with high-flow ventilation, 9 (13.8%) with non-invasive ventilation, 10 (15.4%) with invasive mechanical ventilation, 7 (18.9%) with prone position ventilation, 6 (16.2%) with extracorporeal membrane oxygenation (ECMO), and 3 (8.1%) with renal replacement therapy. Only 1 (2.7%) patient had died in the 28-day follow up in our study. All patients had bilateral infiltrates on their chest CT scan. Twenty-one (32.3%) patients presented ground glass opacity (GGO) with critical patients more localized in the periphery and the center. The mortality of critical patients with the COVID-19 infection is low in our study. Cardiac function was enhanced in the early stage and less likely to develop into acute cardiac injury, but most patients suffered with acute liver injury. The CT imaging presentations of COVID-19 in critical patients were more likely with consolidation and bilateral lung involvement.

## Introduction

Since December 2019, the outbreak of the novel coronavirus that originated in Wuhan has spread to more than 100 countries in Asia, Europe, North America, and the Middle East, and has become a global threat to human health. In February 2020, the World Health Organization designated the disease COVID-19 (1). More than 80,000 people have been infected in China, South Korea, Iran, and Italy, who are coping with significant outbreaks. Many studies have reported the clinical, epidemiological, laboratory, and radiological characteristics, and also treatment and clinical outcomes of patients confirmed with COVID-19 pneumonia (2–5). However, most of those studies focused mainly on Wuhan or Hubei. There are significant regional differences in the outcomes of COVID-19 in Wuhan and elsewhere. The pathophysiology of COVID-19 has gradually been recognized, the mortality of patients with COVID-19 in Wuhan was significantly higher than that in other regions. Understanding the clinical characteristics of patients in other regions outside Wuhan is really necessary for implementing different levels of prevention and control measures.

By February 17, 2020, there were 504 confirmed cases reported according to a government announcement in Wenzhou. Yang.et al presented the clinical characteristics and chest CT scan manifestations of most of the mild patients in Wenzhou, but did not report the severe COVID-19 characteristics (6). Aimed at exploring clinical characteristics and outcomes of hospitalized severe or critical patients with confirmed COVID-19 infection in Wenzhou, here, we show details of those patients admitted to the First Affiliated Hospital of Wenzhou Medical University and clinical outcome as of 28-day mortality.

## Materials and Methods

### Study Design and Participants

For this single-centered, retrospective cohort study, we recruited adult inpatients (≥18 years old) admitted to The First Affiliated Hospital of Wenzhou Medical University, the only designated hospital in Wenzhou for patients with a severe or critical COVID-19 infection. The patients were consecutively enrolled from January 28 to February 16. Diagnosed with COVID-19 according to a laboratory reverse transcription polymerase chain reaction (RT-PCR) test, all these adult patients were confirmed as having the COVID-19 infection. The study was approved by The First Affiliated Hospital of Wenzhou Medical University Ethics Committee.

### Data Collection

We retrospectively collected the medical records which included epidemiological, demographic, symptoms, laboratory results, complications, outcome, and treatment data. We collected the admission data of these patients. Laboratory confirmation of COVID-19 was done by real-time RT-PCR methods.

The data on age, sex, exposure history, comorbidity (hypertension, diabetes cardiovascular disease, chronic kidney disease, chronic liver disease, cerebrovascular disease, hematological diseases), symptoms from onset to hospital admission (fever, cough, expectoration, dyspnea, muscle pain, headache, sore throat, chill, diarrhea, fatigue), laboratory results on admission (hemoglobin, white blood cell count, neutrophil count, lymphocyte count, monocyte count, platelet, d-dimer, creatine kinase, creatine kinase–mb, alanine aminotransferase, aspartate aminotransferase, total bilirubin, blood urea nitrogen, creatinine, hypersensitive troponin I, procalcitonin, brain natriuretic peptide, lactate, albumin, total cholesterol, cytokine levels, T lymphocyte cell subsets), treatment [Glucocorticoid therapy, Immunoglobulin therapy, Thymosin, Thaliduan, Antibiotic treatment, Antiviral treatment, Oxygen Treatment, Prone Position Ventilation, Continuous renal replacement therapy (CRRT)], clinical outcome [Sepsis, Septic Shock, Acute respiratory distress syndrome (ARDS), Acute cardiac injury, Acute kidney injury, Acute liver injury, Secondary infection, Acidosis, Prognosis], and radiological and echocardiography data and as well as living status were collected. The Acute Physiology and Chronic Health Evaluation II (APACHEII) and SOFA score system were used to assess pneumonia severity.

### Definitions

Sepsis and septic shock were diagnosed according to sepsis-3.0 definition (7). Acute kidney injury was defined according to the KDIGO clinical practice guidelines; acute cardiac injury was diagnosed if serum levels of cardiac biomarkers (e.g., high sensitive cardiac troponin I or brain natriuretic peptide) increased. Acute respiratory distress syndrome (ARDS) was defined by the Berlin definition. Acute liver injury was diagnosed if serum levels of liver biomarkers (e.g., Alanine aminotransferase, Aspartate aminotransferase, Total bilirubin) increased. Secondary infection was defined when patients had a positive culture of a new pathogen after admission. The disease severity (severe or critical) was defined according to the 6th edition guideline issued by China's National Health Commission (7).

### Statistical Analysis

Continuous variables were presented as medians (IQR) or mean (SD) and compared with the Mann–Whitney *U*-test. Categorical variables were presented as *n* (%) and were compared by the chi-square or Fisher exact test between severe and critical patients with COVID-19. We used SPSS (version 22.0) for all analyses. A *p* < 0.05 was considered as statistically significant, statistical analyses were done using the SAS software, version 24.0.

## Results

By February 16, 2020, 37 severe or critical patients had been admitted to The First Affiliated Hospital of Wenzhou Medical University with confirmed COVID-19 pneumonia, of whom, 15 (40.5%) severe and 22 (59.5%) critical. The mean age was 57 years (21–93), 14 (21.5%) patients were older than 60 years old. Twenty-six (68.4%) patients were men. Ten (26.3%) patients recently visited Wuhan or Hubei, and 13 (34.2%) had contact with Wuhan residents, 4 (10.5%) had been exposed to a confirmed case, 11 (28.9%) patients had no definite causes. The most common chronic diseases were hypertension [14(36.8%)] and diabetes [8(21.1%)]. Twenty-two patients had increased serum levels of aspartate aminotransferase. All of the 37 patients had bilateral infiltrates on their chest CT scan. The most common symptoms were fever (100%), cough (78.4%), and expectoration (43.2%). The median APACHE II score and SOFA score of all patients were 8.0 (4.5–10.5) and 1 (0–3) (detail in Table 1).

**Table 1:** Baseline characteristics of patients infected with COVID-19.

**Variable**	**ALL patients (*n* = 37)**	**Severe (*n* = 15)**	**Critically severe (*n* = 22)**	***p***
**Clinical parameters**				
Age, median (IQR), year	55 (48–68)	54 (48–60)	56 (47–73)	0.132
>60 year	14 (21.5%)	3 (20.0%)	10 (45.5%)	0.111
Male gender, *n* (%)	26 (68.4%)	11 (73.3%)	15 (76.2%)	0.736
hospital-stay, mean (SD), d	30.4 (14.7)	25.1 (15.8)	31.9 (11.1)	0.134
**Exposure history no. (%)**				
Recently visited Wuhan or hubei	10 (26.3%)	4 (26.7%)	6 (27.3%)	0.967
Contact with Wuhan residents	13 (34.2%)	7 (46.7%)	6 (27.3%)	0.225
Exposure to patients^*^	4 (10.5%)	1 (6.7%)	3 (13.6%)	0.503
No definite causes	11 (28.9%)	4 (26.7%)	7 (31.9%)	0.875
**Comorbidity no. (%)**				
Hypertension	14 (36.8%)	6 (40.0%)	8 (36.4%)	0.823
Diabetes	8 (21.1%)	3 (20%)	5 (36.4%)	0.843
Cardiovascular disease	1 (2.6%)	0	1 (2.6%)	–
Chronic kidney disease	1 (2.6%)	0	1 (2.6%)	–
Chronic liver disease	1 (2.6%)	0	1 (4.5%)	–
Cerebrovascular disease	1 (2.6%)	0	1 (2.6%)	–
Hematological diseases	1 (2.6%)	0	1 (2.6%)	–
**Signs and symptoms no. (%)**				
Fever	37 (100%)	15 (100%)	22 (100%)	–
Cough	29 (78.4%)	11 (73.3%)	18 (81.8%)	0.835
Expectoration	16 (43.2%)	6 (40%)	10 (45.5%)	0.742
Dyspnea	8 (21.6%)	2 (13.3%)	6 (27.3%)	0.545
Muscle pain	4 (10.8%)	2 (13.3%)	2 (9.1%)	1.000
Headache	2 (5.4%)	2 (13.3%)	0 (0%)	0.158
Sore throat	4 (10.8%)	3 (20%)	1 (4.5%)	0.344
Chill	8 (21.6%)	5 (33.3%)	3 (13.6%)	0.307
Diarrhea	2 (5.4%)	1 (6.7%)	1 (4.5%)	1.000
Fatigue	7 (18.9%)	2 (13.3%)	5 (22.7%)	0.773
**Scoring sytems**				
APACHEII, median (IQR)	8 (4.5–10.5)	7 (4–9)	10 (6–13)	0.042
SOFA, median (IQR)	1 (0–3)	0 (0–2)	2 (1–3)	0.012

APACHEII, The median Acute Physiology and Chronic Health Evaluation II; SOFA, sequential organ failure assessment; Data are n (%) or mean (SD) median (IQR); patients,

* *Patients who have confirmed COVID-19 infection or are highly suspected of being infected*.

Most patients had organ dysfunction during hospitalization, including 11 (29.7%) with ARDS, 8 (21.6%) with acute kidney injury, 17 (45.9%) with acute cardiac injury, and 33 (89.2%) with acute liver dysfunction. Only 3 patients (8.1%) had hypersensitive troponin I increased on admission. Eighteen (48.6%) patients were treated with high-flow ventilation, 9 (13.8%) with non-invasive ventilation, 10 (15.4%) with invasive mechanical ventilation, 7 (18.9%) with prone position ventilation, 6 (16.2%) with extracorporeal membrane oxygenation (ECMO), 3 (8.1%) with renal replacement therapy. 37 (100%) received antibacterial agents and antiviral treatment, 21 (56.8%) patients received glucocorticoids. Thymosin was treated in 21 (56.8%) of the patients. Thaliana was treated in 15 (40.5%) of the patients (Details in Tables 2, 3).

**Table 2:** Laboratory findings of patients infected with COVID-19 on admission to ICU.

**Variable**	**Normal range**	**ALL patients (*n* = 37)**	**Severe (*n* = 15)**	**Critically severe (*n* = 22)**	***p***
Hemoglobin, g/L	115–150	127.0 (112–142)	128.0 (119–150)	126.0 (109–139)	0.202
White blood cell count, × 10^9^/L	3.5–9.5	7.8 (5.3–11.4)	5.6 (4.8–8.0)	9.3 (6.9–11.8)	0.135
Neutrophil count, × 10^9^/L	1.8–6.3	6.1 (3.3–9.4)	4.8 (2.4–7.1)	7.0 (4.6–9.6)	0.197
Lymphocyte count, × 10^9^/L	1.1–3.2	0.7 (0.5–1.0)	0.8 (0.6–1.3)	0.7 (0.5–1.0)	0.449
Monocyte count, × 10^9^/L	0.1–0.6	0.5 (0.3–0.7)	0.4 (0.3–0.7)	0.5 (0.3–0.9)	0.266
Platelet count, × 10^9^/L	125–350	207.0 (114–259)	253.0 (191–289)	179.0 (106–252)	0.005
D-dimer, mg/L	0–0.5	1.1 (0.7–1.7)	1.0 (0.7–1.2)	1.2 (0.8–1.8)	0.329
Creatine kinase, U/L	20–140	129.0 (63–258)	90.0 (52–244)	152.5 (69–355)	0.111
Creatine kinase–MB, U/L	0–16	9.0 (7–13.5)	8.0 (6–10)	11.0 (7.8–12.3)	0.062
Alanine aminotransferase, U/L	7–40	31.0 (21.5–61.5)	29.0 (24–42)	38.5 (20.8–69.5)	0.596
>40, *N* (%)		16 (43.2%)	5 (33.3%)	11 (50.0%)	0.315
Aspartate aminotransferase, U/L	13–35	41.0 (30.5–59.0)	35.0 (30–44)	50.0 (34.2–69.0)	0.060
>35, *N* (%)		22 (57.9%)	6 (40%)	16 (72.7%)	0.047
Gamma-glutamyl transferase, U/L	10–60	43.0 (26.0–96.5)	33 (24–95)	49.5 (26.8–101.0)	0.284
>60, *N* (%)		16 (43.2%)	5 (33.3%)	10 (45.5%)	0.460
Total bilirubin, mmol/L	0–20	13.0 (9–16)	14.0 (9–15)	11.5 (8.0–16.3)	0.655
Blood urea nitrogen, mmol/L	2.5–6.1	5.0 (3.9–6.0)	4.0 (3–5.1)	5.24 (4.4–6.9)	0.018
Creatinine, μmol/L	46–92	60.0 (55–76)	57.0 (52–63)	64.5 (57.5–80.8)	0.044
Hypersensitive					
troponin I, ug/L ≥0.015, No. (%)	0–0.015	3 (8.1%)	0	3 (13.6%)	0.202
Procalcitonin, ng/mL ≥0.05, No. (%)	<0.05	2 (5.4)	1 (6.7%)	1 (4.5%)	0.951
Brain natriuretic peptide, pg/ml	0–100	39.0	31.0 (14–58)	64.5 (15.0–156.0)	0.056
Lactate, mmol/L	0.7–2.1	2.4 (1.9–3.1)	2.8 (2.4–3.1)	2.2 (1.7–3.0)	0.143
Albumin, g/l		31.9 (29.1–34.6)	32.8 (30.5–34.9)	30.5 (28.5–33.9)	0.279
Total cholesterol, mmol/l		3.9 (3.5–4.6)	4.4 (3.7–5.2)	3.8 (3.4–4.4)	0.075
IL-2, pg/ml	<3.1	0.9 (0.6–1.1)	0.9 (0.6–1.1)	0.9 (0.7–1.1)	0.357
IL-4, pg/ml	<3.0	0.8 (0.6–1.4)	1.0 (0.5–1.4)	0.7 (0.6–1.4)	0.843
IL-6, pg/ml	<3.0	12.7 (3.49–80.2)	4.9 (2.5–52.4)	38.6 (4.6–103.1)	0.115
IL-10, pg/ml	<4.1	5.9 (3.6–14.6)	4.3 (3.1–7.1)	11.4 (3.7–18.5)	0.367
TNF-a, pg/ml	<3.1	0.2 (0.1–0.5)	0.2 (0.1–0.5)	0.2 (0.1–0.5)	0.716
T cell(CD3+), %	53.7–80.9	53.6 (42.5–66.3)	53.6 (41.3–64.1)	54.3 (42.5–67.9)	0.952
CD4+T cell, %	27.4–49.2	32.3 (27.8–40.1)	32.2 (28–40.2)	33.3 (27.4–40.3)	0.962
CD8+T cell, %	15.8–37.5	18.3 (12.5–27.1)	18.3 (10.9–28.0)	18.3 (13.4–24.5)	0.910
B cell, %	5.1–20.3	22.2 (14.2–29.4)	20.7 (12.8–24.9)	23.9 (14.8–40.8)	0.167
NK cell, %	6.7–30.9	13.9 (10.8–27.8)	18.7 (13.8–28.5)	12.9 (7.7–19.1)	0.069

*Data are n (%) or median (IQR)*.

**Table 3:** Treatments and outcomes of patients infected with COVID-19.

**Variable**	**ALL patients (*n* = 37)**	**Severe (*n* = 15)**	**Critically severe (*n* = 22)**	***P***
**Treatment**				
Glucocorticoid therapy	21 (56.8%)	3 (20%)	18 (81.8%)	<0.001
Immunoglobulin therapy	19 (51.4%)	7 (46.7%)	12 (54.5%)	0.638
Thymosin	21 (56.8%)	4 (26.7%)	17 (77.3%)	0.002
Thaliduan	15 (40.5%)	4 (26.7%)	11 (50%)	0.156
Antiviral treatment	37	15	22	–
Antibiotic treatment	37	15	22	–
**Oxygen treatment**				
High flow	18 (48.6%)	0	18 (81.8%)	<0.001
NIV	9 (13.8)	0	9 (40.9%)	0.005
IMV	10 (15.4%)	0	10 (45.5%)	0.002
ECMO	6 (16.2%)	0	6 (27.3%)	0.063
Prone Position Ventilation	7 (18.9%)	0	7 (31.8%)	0.028
CRRT	3 (8.1%)	0	3 (13.0%)	–
**Complications**				
Sepsis	34 (91.9%)	12 (80%)	22 (100%)	0.059
Septic Shock	6 (16.2%)	0	6	0.063
ARDS	11 (29.7%)	0	11 (50%)	<0.001
Acute cardiac injury	17 (45.9%)	1 (6.7%)	16 (72.7%)	<0.001
Acute kidney injury	8 (21.6%)	1 (6.7%)	7 (31.8%)	0.156
Acute liver injury	33 (89.2%)	12 (80.0%)	21 (95.5%)	0.344
Secondary infection	13 (35.1%)	1 (6.7%)	12 (54.5%)	0.003
Acidosis	10 (27.0%)	0	10 (45.5%)	0.002
**Prognosis**				
28-day mortality	1 (2.7%)	0	1 (4.5%)	

*Data are n (%); NIV, non-invasive ventilation; IMV, invasive mechanical ventilation; ECMO, extracorporeal membrane oxygenator; CRRT, continuous renal replacement therapy; ARDS, Acute respiratory distress syndrome*.

Among the 37 severe or critical patients with the COVID-19 infection, only 1 (2.7%) patient died, after 14 days. Compared with severe patients, critical patients were older [52.4 (21–81) vs. 60.1 (39–93)], and had high APACHE II [7 (4–9) vs. 10 (6–13)], and SOFA scores [0 (0–2) vs. 2 (1–3)]. Of the 22 critical patients, 13 (59.1%) patients were discharged, 8 (36.4%) patients remained in hospital (Details in Table 3)

The radiological and echocardiography data on admission are summarized in Table 4. All patients had bilateral infiltrates on their chest CT scan. Twenty-one (32.3%) patients presented ground glass opacity (GGO) with critical patients more localized in the periphery and the center. Thirty patients had echocardiography data. The most common abnormality in echocardiography was diastolic dysfunction and left atrial enlargement. All cases had normal cardiac output or increased cardiac output.

**Table 4:** Radiological and echocardiography data of patients infected with COVID-19.

**Variable**	**ALL patients (*n* = 37)**	**Severe (*n* = 15)**	**Critically severe (*n* = 22)**
**Density**			
GGO	21 (32.3%)	11 (66.7%)	10 (45.5%)
consolidation	1 (1.5%)	0	1 (4.5%)
mixed	15 (23.1%)	4 (27.6%)	11 (50.0%)
**Location**			
Peripheral	15 (40.5%)	9 (60.0%)	6 (27.3%)
Central and peripheral	22 (59.5%)	6 (40.0%)	16 (72.7%)
Pleural effusion	3 (4.6%)	2 (13.3%)	1 (4.5%)
Echocardiography, *N*	30	10	20
cardiac output, CO L/min	6.0 (1.6)	6.6 (1.6)	5.7 (1.5)
stroke volume, SV ml	75.4 (16.7)	79.6 (18.7)	73.4 (15.6)
Ejection fraction, EF %	63.5 (4.8)	66.0 (3.5)	62.3 (5.0)
Diastolic dysfunction	17 (56.7%)	7 (70%)	10 (50%)
pulmonary hypertension	2 (6.7%)	0	2 (10%)
Left atrial enlargement	17 (56.7%)	5 (50%)	12 (60%)
Mitral regurgitation	7 (23.3%)	0	7 (35%)
Tricuspid regurgitation	4 (13.3%)	0	4 (20%)

*GGO, ground glass opacity; Data are n (%)*.

## Discussion

This retrospective cohort study presented severe and critical patients' clinical characteristics and outcomes in Wenzhou city who were hospitalized with the COVID-19 infection. In this study, we found higher age, and higher SOFA and APACHE II scores on admission were associated with disease severity. Additionally, elevated levels of Blood urea nitrogen, decreased levels of platelet were more common in critical COVID-19 patients. To our best knowledge, this retrospective cohort study is the first report to compare severe and critical patients from one city with COVID-19 outside Wuhan.

Comparing our data with those published from Wuhan, we found that patients in Wenzhou city had a milder infection (8, 9). According to our study, the epidemiological, demographic, symptom, and laboratory results, and CT scans of COVID-19 were similar to these previous studies (6, 7, 9). The announced mortality in Hubei is much higher, which shows a significant regional difference. Compared with female or those of a younger age, male, and people of an older age (>65 years) are more likely to develop ARDS (10). In previous studies, evidence found that older and male patients are the most susceptible to the COVID-19 infection, nearly 70% of patients infected by COVID-19 were male (7, 11), which is also supported by our data. We observed that critical patients were significantly older than severe patients. Elderly patients experienced a markable decline in cell-mediated immune function and reduced human immune function. The induction of proinflammatory cytokines after infection is not adequately controlled by anti-inflammatory mechanisms in elderly persons, potentially leading to a poor prognosis (12).

Liver complications, including elevated levels of ALT, AST, or bilirubin are common in patients with sepsis. Several large-scale case studies have reported the clinical features of patients with COVID-19 (4, 6, 9). These data indicate that 14–53% of patients with COVID-19 reported increased levels of ALT and AST during disease progression. Patients with severe or critical COVID-19 seem to have higher rates of liver dysfunction. In our study, elevation of ALT or AST was observed in 22 (33.8%) out of 37 patients. In addition, gamma-glutamyl transferase (GGT), which is a diagnostic biomarker for cholangiocyte injury, has not been reported clearly. We found that GGT was elevated in 26 (70.3%) out of 37 patients with COVID-19 during hospitalization. Compared with severe cases, critical cases were more likely to have an elevation of AST, liver injury was also related to the disease severity. Angiotensin-converting enzyme 2 (ACE-2) is a membrane-bound aminopeptidase that has a vital role in the liver and immune systems (13). ACE-2 has been identified as a functional receptor for coronaviruses (14). The preliminary study by Chai et al. suggested that ACE-2 receptor expression is enriched in cholangiocytes, indicating that COVID-19 might directly bind to ACE-2-positive cholangiocytes to dysregulate liver function (15).

Based on previous studies, nearly 17% of patients had acute cardiac injury, with a high sensitive cardiac troponin I increase or abnormalities seen in electrocardiography and echocardiography (4, 8, 11). In our study, only 3 critical patients had hsCnTI mile increase on admission, showing a significant regional difference. Cardiac function was enhanced in the early stage and was less likely to develop into acute cardiac injury. The potential explanation of this difference may be early intervention. For critical patients, COVID-19 manifests as “silent hypoxemia,” showing rapid deterioration and death after admission, which may begin the process of long-term hypoxia which can easily cause damage to myocardial cells. In Wenzhou, the government searched for suspected COVID-19 patients to be admitted to the hospital for treatment, we were capable enough to provide effective medical care to all infected patients to improve the prognosis of COVID-19.

The APACHEII and SOFA scores reflect the state and degree of illness severity and multi-organ dysfunction, respectively, the SOFA score is also a good diagnostic marker for sepsis (16, 17). High APACHEII and SOFA scores on admission can help clinicians to identify a patient's illness severity at an early stage. In previous studies, more than half of the patients developed sepsis. In addition, we found that sepsis is really common in severe and critically ill patients, but only several developed septic shock. Decreases in CD4+ T-cell levels, and lymphopenia and abnormal cytokine levels were common features in cases of COVID-19, which might be a critical factor associated with disease severity and mortality (18, 19). We observed a decrease in lymphocytes and a significant increase in cytokine IL-6 levels, but in our case cohort, only a few patients' CD4+ T-cell levels decreased. It shows that those patients had a milder overall condition and they also may have benefited from receiving timely and effective intervention and treatment, and their immune levels had not significantly decreased. Furthermore, more than half of the patients received immunoglobulin and Thymosin therapy to improve immunity.

Our study has some limitations. Firstly, several patients in our study still remain in the hospital, the final outcome is not complete, but those patients have survived longer than 28 days. Secondly, several patients were transferred from other medical institutions and may have received effective intervention before being transferred, this hospital admission may not be their first admission. Thirdly, only 37 patients were included, our conclusion might be limited by the sample size.

## Conclusions

The mortality of critically ill patients with COVID-19 is low in our study. Cardiac function was enhanced in the early stage and therefore less likely to develop into acute cardiac injury, but most patients suffered with acute liver injury. The CT imaging presentations of COVID-19 in critical patients were mostly patchy ground glass opacities in the peripheral areas under the pleura, and more likely with consolidation, and bilateral lung involvement.

## Data Availability Statement

The raw data supporting the conclusions of this article will be made available by the authors, without undue reservation.

## Ethics Statement

The study was approved by the First Affiliated Hospital of Wenzhou Medical University Ethics Committee.

## Author Contributions

J-YP: design of the study. S-ZQ and WH: collection of data. L-m: data management. C-l and Z-f: analysis. S-ZQ: wrote the paper. J-YP: critical revision of the article. All authors contributed to the article and approved the submitted version.

## Conflict of Interest

The authors declare that the research was conducted in the absence of any commercial or financial relationships that could be construed as a potential conflict of interest.
